# COVID-19 vaccine hesitancy in conflict zones: A review of current literature

**DOI:** 10.3389/fpubh.2022.1006271

**Published:** 2022-12-02

**Authors:** Amna Siddiqui, Priya, Alishba Adnan, Samina Abbas, Khulud Qamar, Zarmina Islam, Zainab Syyeda Rahmat, Mohammad Yasir Essar, Ramadan Abdelmoez Farahat

**Affiliations:** ^1^Department of Medicine, Karachi Medical and Dental College, Karachi, Pakistan; ^2^Department of Medicine, Dow University of Health Sciences, Karachi, Pakistan; ^3^Afghanistan National Charity Organization for Special Diseases, Kabul, Afghanistan; ^4^Kabul University of Medical Sciences, Kabul, Afghanistan; ^5^Faculty of Medicine, Kafrelsheikh University, Kafr El-Shaikh, Egypt

**Keywords:** COVID-19, vaccine hesitancy, conflict zones, Afghanistan, Yemen, Syria, Palestine, Somalia

## Abstract

**Background:**

Vaccine hesitancy (VH) is prevalent in conflict zones due to a lack of essential resources and knowledge, thereby escalating the coronavirus disease of 2019 (COVID-19) cases in these territories. This has resulted in a higher incidence of cases from exposure to a single COVID-19 positive case and further burdens the health care system of conflict zones which are already on the brink of collapsing.

**Aim:**

This narrative review aims to determine VH to the severe acute respiratory syndrome coronavirus 2 (SARS-CoV-2) vaccine in five conflict zones that include Somalia, Yemen, Palestine, Syria, and Afghanistan.

**Methodology:**

A Boolean search was carried out in MEDLINE-PubMed from inception till 6 June 2022. The search was performed by using the following keywords: “(SARS-CoV-2 OR covid OR covid 19) AND (vaccine hesitancy OR covid vaccine acceptance OR intention to vaccinate) AND (Syria OR Yemen OR Palestine OR Afghanistan OR Somalia”). The full text of all relevant articles in English along with their supplementary material was extracted.

**Results:**

All the included studies reported at least 30% or more increase in vaccine hesitancy among conflict settings. VH was mostly due to a lack of available resources, lack of appropriate knowledge, and believing misleading rumors about the vaccine.

**Discussion:**

Considering the massive amount of reluctance among people residing in conflict zones, the need to take effective measures against VH is undoubtedly apparent. This can be accomplished by carrying out mass vaccinations by the governments and proper health education through raising the public awareness regarding vaccines, thereby eliminating rumors that exacerbate the fear of adverse effects.

**Conclusion:**

The approach described in this article to combat VH can be implemented to increase vaccination rates and significantly alleviate R_0_ across the globe.

## Introduction

Vaccination is a widely utilized strategy for most countries to repress Coronavirus disease of 2019 (COVID-19), also known as severe acute respiratory syndrome coronavirus 2 (SARS-CoV-2) transmission. It has been the best identified cost-effective approach, which primarily aims to provide potent and long-term immunity against the COVID-19 infection (1). COVID-19 is a zoonotic air-borne infection, transmitted *via* human-to-human, which has regional variations in its incidence and prevalence. Hence, explaining the reason behind uneven implementation of the two most effective strategies which are comprehensive intervention and global lockdown. Second, although it is dynamic, the basic reproduction number (R_0_/R naught) is an epidemiologic entity that helps predict the expected number of cases from exposure to a single case, assuming all the individuals in the given population are susceptible. Further, the pooled R_0_ of the meta-analysis by Zhang et al. (2), specifies there should be at least 93.3% of people being vaccinated around the world to efficiently control the COVID-19 pandemic. Therefore, the inadequate response of many countries to quell the pandemic successfully is because currently only around 65% of people are vaccinated around the world (3). Thus, vaccine hesitancy (VH) or reluctance to get vaccinated in a specific region markedly forms a critical barrier to competently combat the disease, as evidenced by subsequent increase in COVID-19 cases and associated disease-specific mortality rates in the respective territories (4, 5).

COVID-19 has exacerbated the world's worst humanitarian disasters and propelled undercurrents, leading some countries to strain economically to a breaking point. The consequence of this economic mayhem is civil uproar, as demonstrated by civilians around the world leaping from discontent to protest, from protest to crisis, and eventually from crisis to conflict. Long-standing conflict has shown to instigate disruption in essential services, such as housing, transportation, communication, sanitation, water, and healthcare and may require global intervention from people outside of the affected community (6). Moreover, there is an innate mistrust or distrust of information i.e., on COVID-19 and vaccines that are deployed in such zones, possibly due to political instability in the state. Subsequently, conflict-settings observes increased VH relative to conflict-free settings, thereby escalating the R_0_ of COVID-19 cases and adding an additional burden on the debilitated health care of conflict zones.

The current study considers five countries (i.e., Afghanistan, Somalia, Syria, Palestine, and Yemen), based on the record of ongoing conflicts. They possess remarkably lower rates of vaccine acceptance as provided in the survey study by Sallam et al., conducted from 2020 to 2021 and found it to be lower than almost 80% (7, 8) relative to an overall global acceptance rate that varies across countries, ranging from 35.9 to 86.9% for adults on an average (9). Thus, considering the significance of vaccinating large cohorts to alleviate the R_0_ of COVID-19 cases across the globe, our narrative review aims to provide a concise perspective and report on the status of COVID-19 VH rates in the conflict settings mentioned to highlight the devastating effect that VH has in the conflict zones to add to the literature. Further, the insight provided in this article to tackle VH can be implied to increase vaccination rates and significantly alleviate R0 for COVID-19 cases across the globe, subsequently curbing the pandemic in a pragmatic way.

## Methodology

**Figure 1:**
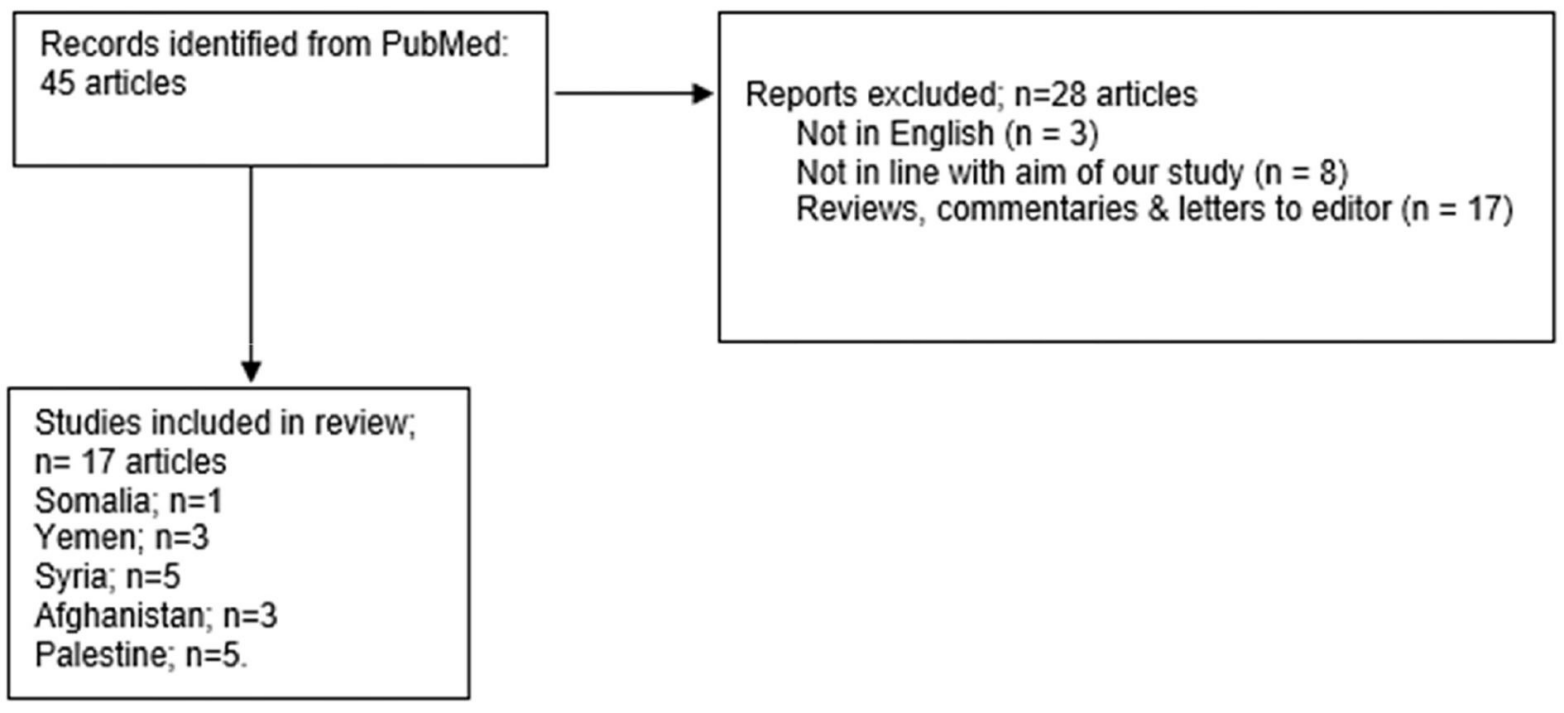
Flowchart displaying the screening process for the included studies in the review.

A Boolean search was carried out in MEDLINE-PubMed, from inception till June 6, 2022, using the search string “(SARS-CoV-2 OR COVID OR COVID 19) AND (vaccine hesitancy OR covid vaccine acceptance OR intention to vaccinate) AND (Syria OR Yemen OR Palestine OR Afghanistan OR Somalia)”. Full text of all the related articles in English with supplementary appendices was retrieved. Additionally, the full text of relevant cross-references was also retrieved. The articles retrieved are enlisted in Table 1. The Inclusion criteria for studies were as follows: (1) Freely accessible, full articles (2) Original studies, observational, cross-sectional, and randomized controlled trials (3) Papers analyzing vaccine hesitancy among cohorts of conflict zones (i.e., Syria, Yemen, Palestine, Afghanistan, and Somalia) and (4) Only Published studies in peer-reviewed journals were included for cohorts in stated conflict settings. However, all the reviews, editorials, commentaries, case reports, and case series were excluded. A total of 17 studies were included in our review, details of the screening process are displayed in the flowchart below (Figure 1).

**Table 1:** Summary of studies on vaccine hesitancy in cohorts in conflict settings (i.e., Somalia, Yemen, Syria, Afghanistan, and Palestine).

**Author**	**Methodology**	**Results**
**Somalia**		
1. Ahmed et al. (10)	4,543 General adult population. An electronic survey link was circulated on the social media platforms of Mogadishu University	Vaccine acceptance is relatively high but could be improved by addressing causative factors. 76.8% were willing to receive a COVID-19 vaccine, 283 were concerned about inefficacy, and 424 feared side effects. 204 defended that only strong natural immunity matters. 308 believed the pandemic to be over
**Yemen**		
1. Bitar et al. (11)	484 participants filled out an online questionnaire having 4 main sections	The acceptance rate was suboptimal and significantly affected by gender (men were more accepting), misinformation, cost, and income The study also revealed that 61.2% would take the vaccine if it was free and 43% would take the vaccine if it wasn't free
2. Noushad et al. (12)	5,329 participants filled an online questionnaire shared *via* WhatsApp	Severe shortage and lack of access to vaccines, rather than vaccine hesitancy 50.1% were willing. 66.4% were worried about side effects
3. Bin Ghouth et al. (13)	Health care workers and other general populations were interviewed *via* verbal consent	Lack of knowledge about the COVID-19 vaccine and a high level of vaccine hesitancy were reported. The most common reasons were: bad quality of the vaccine and doubts about vaccine safety
**Syria**		
1. Kaadan et al. (14)	870 participants. General adult population Vaccine acceptance tendencies were collected and analyzed using Chi-squared (χ^2^) test and Logistic regression	63.6% accepted vaccination. Among 22 Arab countries, < 66% were compliant to get vaccinated, this result falls lower compared to global levels
2. Shibani et al. (15)	7,531 participants. Health care workers & Social media users. Vaccine acceptance tendencies were collected and analyzed using Chi-squared (χ^2^) test and Logistic regression	37% were certain to get vaccinated, as during this study the vaccines had not reached Syria. Results mostly included higher-income communities (more hesitant) due to their accessibility to the internet. 31% were uncertain due to: fear of side effects (62.4%), and mistrust of the vaccine formula (58.8%)
3. Zein et al. (16)	8,619 people from the general adult population Vaccine acceptance tendencies were collected and analyzed using Cross tabulations and *y*2 tests	Mainly middle-aged adults and undergraduates were hesitant to be vaccinated. Reasons were lack of knowledge, exposure to anti-vaccine content on social media, and mistrust in health care facilities. 32.2% were willing, 41.6% not willing, and 26.2% were not sure
4. Mohamad et al. (17)	1,222 general adult population. Vaccine acceptance tendencies were collected and analyzed using multivariate analysis	35.92% were willing. The most common reasons for vaccine hesitancy were fear of side effects, doubts about vaccine efficiency, timing, importance, and just simple refusal
5. Noushad et al. (18)	2,963 health care workers & social media users Vaccine acceptance tendencies were collected and analyzed using Bivariate statistical analyses, Chi-squared test and multivariate analysis	Many L-LMICs did not have access to the vaccine whilst this study was conducted leading to vast misconception and increased hesitancy toward the vaccine Vaccine acceptability was 69%. HCWs from UM-HICs are more willing to get vaccinated compared to those from L-LMICs (75 vs. 62%). Older HCWs were more willing compared to younger ones
**Afghanistan**		
1. Nemat et al. (19)	806 people have internet access. The survey was shared *via* social media and telegram channels	A significant portion of the public was not willing to take the COVID-19 vaccine. Less than 63% reported willingness to take it once it becomes available and 37% were hesitant
2. Chen et al. (20)	Afghan healthcare providers (*n* = 224). Online survey	Hesitancy was calculated by (MoVac-COVID19S) and the validity of this scoring system was judged
3. Ahorsu et al. (21)	Jamhuriat hospital, Kabul, Afghanistan. 6,053 participants across the included countries completed the survey. Survey (on paper or online)	Hesitancy was calculated by (MoVac-COVID19S) and data were analyzed by IBM SPSS 22.0
**Palestine**		
1. Maraqa et al. (22)	1,159 healthcare workers filled out an online survey	They had doubts about long-lasting immunity, the possibility of catching COVID-19 from the vaccine, efficacy, long-term and short-term side effects of the vaccine, if it is painful and mostly fostered the misinformation about it
2. Kateeb et al. (23)	417 Palestinian dental students, WHO SAGE Vaccination Hesitancy Questionnaire	Reasons for hesitancy were the availability of the vaccine, trust in pharmaceutical companies, and thinking that their natural immunity will suffice
3. Belkebir et al. (24)	46 nurses working in government and private facilities filled an online or a paper survey	They were uncertain as they doubted if the COVID-19 virus would mutate or not, how long would it take for vaccine development, had questions about types of vaccines, and their timing for doses. This was mainly due to a lack of education and mass media misinformation in the state
4. Rabi et al. (25)	639 Palestinian nurses, filled out a self-administered questionnaire	Reasons for hesitancy were age, lack of knowledge about the vaccine, fear of long-term side effects and getting injected, believing more in the strength of their natural immunity, being influenced by media misinformation, and the possibility of getting COVID-19 from the vaccine
5. Al-Kafarna et al. (26)	6,226 general Palestinian participants in the Gaza strip and the West Bank, filled out an online questionnaire	Mistrust in; (1) Pharmaceutical companies and their attempts at making a profit off this, (2) Benefits of the vaccine, (3) Vaccines are made anywhere apart from the USA or Europe. Fear of side effects and believing that their natural immunity is better

## Challenges and breakthrough: A review of vaccine hesitancy in conflict settings

The studies retrieved through the screening process (Figure 1) are summarized in Table 1. It enlists the summary of studies based on analyzing vaccine hesitancy in cohorts located in conflict settings (i.e., Somalia, Yemen, Syria, Afghanistan, and Palestine). The results are thematically elaborated specific to conflict settings relative to the possible determinants for the findings quoted in Table 1.

### Personal beliefs

There were three studies published in Afghanistan for the analysis of VH in Afghani population. The study details including the results are summarized in Table 1.

According to the study by Nemat et al. (19), most of the participants around eight-tenths were aware of the efforts by the government to develop vaccines for COVID-19, showing their interest in keeping up to date on news on COVID-19. One out of five people doubted the availability of the vaccine in 2021. Sixty-three percent of the participants were willing to take the COVID-19 vaccine upon its availability. A significant correlation was observed in gender association with willingness to get vaccinated with females, more willing to take the COVID-19 vaccine compared to males (77.3 vs. 57.7%; *p* = 0.001). Age groups were not associated with willingness to take the COVID-19 vaccine. 37% of the participants were not willing to take the COVID-19 vaccine.

In the study by Ahorsu et al. (21), they compared the same populations as the study mentioned previously, and there were 224 Afghanis included in this study as well. However, participants were recruited by an online survey shared on social media to collect information from the general population instead of just healthcare workers. The duration of the study stretched between January and March 2021. Only 63% of the participants were willing to take the vaccine, which is much less compared to other countries. Variables of socio-economic data, such as age group, education level, and geographical locations did not show any significant association with COVID-19 vaccine acceptance.

Palestinians have often exhibited VH, in the past for influenza and flu vaccines and in current times for the COVID-19 vaccine (22, 23). Several studies have been carried out to determine VH among the Palestinian population, and those that analyzed and reported VH specifically for COVID-19 are summarized in Table 1.

A cross-sectional study conducted in Palestine among 1,159 healthcare workers (HCWs) to assess VH, reported that only 37.8% of the HCWs were willing to get the COVID-19 vaccine, 31.5% had not decided and 30.7% of participants had planned to not get the vaccine (22). Additionally, a survey conducted among Palestinian dental students included 417 participants, the results reported that 14.9% of the students did not plan to take the vaccine, and 27% of individuals displayed feelings of hesitancy toward the COVID-19 vaccine (23). Moreover, a study that included forty-six nurses assessed different parameters for COVID-19 vaccine uptake including trust, uncertainty, and knowledge. Only one-third of the nurses were willing to take the vaccine, and the rest of them lacked trust in the vaccine, fearing contraction of the virus or encountering side effects (24). Another study performed involving a sample size of 639 nurses also reported that 60% of the nurses had concerns regarding the vaccine and were not willing to get the vaccine (25). Furthermore, a study involving 6,226 Palestinian participants reported a generally positive attitude toward vaccination. However, 37.86% of participants were reluctant to get the COVID-19 vaccine administered (26).

The current literature makes it evident that most of the Syrian population tend to hold back from acquiring the vaccines due to uncertainty and fear. VH in Syria is widespread as many scientific pieces of literature investigated in 2021–2022, summarized in Table 1.

In 2021, Shibani et al. conducted a nationwide cross-sectional study that surveyed 7,531 Syrian people on their willingness to take the COVID-19 vaccine. Around 63% of respondents seemed to be unlikely or uncertain to take the vaccine, the majority being fearful of the side effects (15).

Most studies have analyzed that VH prevails in the Middle Eastern countries, especially among middle- and lower-income countries and those in conflict. Whilst many studies have their limitations, the outcomes from Table 1 portray that Syrian populations vary accordingly in vaccine acceptance. As Noushad et al. (18) state amongst the HCWs, the younger population and those with lower socioeconomic status were more hesitant to receive the vaccine. However, among the general adult population, Zein et al. (16) state middle-aged individuals, those with undergraduate education, and mostly females were hesitant to receive vaccines. The survey results had associated many factors related to hesitancy, one of the most common reasons was the mass circulation of false information on side effects and formulations of the vaccine. People find it easier to believe many deaths or worsening prognosis of diseases are linked to receiving the COVID-19 vaccine without any underlying scientific explanation. During the pandemic, many HCWs had been overwhelmed due to the surge of responsibilities, long work hours and high risk of infectious exposure. Consequently, over time HCWs have been unable to provide efficient treatment and medication, resulting in severe mistrust of HCWs by the general population. This too may be a reason for VH. However, it has also been reported that when HCWs assured the Syrian population that the vaccine was beneficial and free of any severe side effects, this increased the rate of vaccine acceptance. Therefore, widespread awareness is necessary to reduce VH.

### Conspiracy theories

According to the current literature, it is evident that conspiracy theories plays a major role in the vaccine hesitancy observed in different conflict zones.

In a study conducted by Nemat et al. (19) in Afghanistan, Participants were hesitant to get vaccinated because of the following reasons: they assumed the vaccine to be of low quality, they believed they had sufficient natural immunity to fight COVID-19, and that the COVID-19 vaccine was not safe.

The public was concerned about the budget constraints, unstable security, and prevalent corruption due to which they thought the distribution of the vaccine would be limited in Afghanistan. The female population was less affected by the conspiratorial theories compared with males, perhaps due to limited exposure, as mostly restricted from working outside home. The population that was not willing to take the vaccine was sufficiently large (37%) (19). Those unwilling could influence those who were willing by spreading misinformation, eventually becoming a hurdle in reaching herd immunity against COVID-19 in Afghanistan (27). In addition, the low acceptance of the COVID-19 vaccine among the Palestinian population was due to a lack of knowledge about the vaccine, fear of contracting the virus after vaccination and experiencing side effects with vaccine administration (22). In the survey involving dental students, factors causing vaccine hesitancy were investigated. It was reported that the factors that impacted participants' willingness for vaccination included social media (*p* = 0.003) and opinions of influencing personalities such as celebrities, political and religious leaders (*p* < 0.001) (23). The study involving 46 nurses, investigated the impact of appropriate knowledge about the vaccine on the participant's opinions. After attending a lecture and gaining sufficient knowledge about the vaccine, one nurse changed her opinion and was willing for vaccination (24). Moreover, misinformation spread through WhatsApp and Facebook further fuelled VH (25).

A large cohort of people (63%) were willing to take it once it became available, according to the study by Nemat et al. (19). Yet, since Afghani population firmly rely on their “strong immunity” and believe that they do not need external support such as vaccines to prevent COVID-19 becomes a possible reason for vaccine hesitancy of the remaining cohort (28, 29). The most influential reason (43% of people believed this) for hesitance/reticence in the study by Ahorsu et al. (21) was the assumption that since this vaccine was available to low-income countries, it would be of low quality, as 39.6% of people feared the safety of the vaccine. Distrust amongst the population regarding the public health ministry on top of fraud cases is another factor driving vaccine hesitancy, making it arduous to acquire herd immunity (30).

Mohamad et al. (17) mentioned a lack of trust in the effectiveness of the vaccine in providing protection; its formulation was another reason to be hesitant among 65.18% of respondents in his cross-sectional study, conducted through an online survey among the Syrian population. Doubting about the efficacy of the vaccine was more common among female youth, especially those who had higher educational status comparatively to ones with low or no knowledge of the vaccine and had a low financial status (15). The population of Syria comprises of youths and young adults who consider their immunity strong enough to dismiss the idea of being vaccinated for COVID-19. Hence, perceiving the vaccine as not important or a priority (15). Kaadan et al. (14) state that generally in Arab countries, those residing abroad were more likely to accept vaccines as native Arabs had a strong cultural influence that hindered them from getting vaccinated. Noushad et al. (18) conducted a cross-sectional study comprising 2,953 HCWs on a global scale, the results mentioned that HCWs from low-earning countries were more hesitant for COVID-19 vaccination than high-earning countries, mainly resonating on a lack of vaccine coverage and supply. Zein et al. had studied the unwillingness of 8,619 Jordanians, Palestinians, and Syrians to receive vaccines which yielded 67% hesitancy. Most communities have been evaluated to either completely deny any beneficial gain from the vaccine or were precautious of its exaggerated side effects.

An online survey conducted in December 2020 and January 2021, only two months before the COVID-19 vaccine deployment in Somalia, revealed a reasonably high acceptance percentage for the COVID-19 vaccination. The researchers reviewed 4,543 replies and found that 3,488 (76.8%) of study participants were willing to receive the COVID19 vaccination once it became accessible. The efficiency of the vaccination, fear of adverse effects, and faith in their own immunity were among the top reasons for respondents' rejection of COVID-19 summarized in Table 1 (10).

In Somalia, skepticism over the quick development and approval of COVID-19 immunizations, as well as whether the vaccines include any non-halal chemicals, is exacerbated by legitimate worries about the negative effects of the Oxford/AstraZeneca vaccine (31).

In Yemen, According to the online survey by Bitar et al., out of 484 responses, there were about 253 people who had a misconception about COVID-19 and its vaccination. Approximately two-fifths believed that humans have created COVID-19 as a biological weapon. The study found that vaccination acceptance was unsatisfactory and was strongly influenced by gender, misinformation, cost, and poverty. These data demonstrate a strong relationship between sensitivity to misconception and readiness to vaccinate (11). Another survey found that out of 321 responses, 241 participants were unwilling to be vaccinated. The most common reasons for vaccination refusal were poor vaccine quality and a lack of knowledge (13). A large majority of individuals conveyed concern about vaccination adverse effects (66.4%) (12). These findings are summarized in Table 1.

Across Yemen, a variety of notions (including myths about COVID-19 immunization) have taken root. The most frequently stated reasons for poor vaccination uptake by key informants in this study were comparable to the findings of a survey done in de-facto authority (DFA)-controlled regions in early 2021 (32). DFA authority, takes an action without strict legal authority, but is recognized as legally valid, nonetheless. Further, some participants in that study also saw the vaccination as a planned “scheme” that posed harmful to their health. The following were the primary causes and impressions expressed in key informant interviews. Some individuals feel that the vaccination will cause death over time, rather than instantly. Some claim that the vaccine effort is a plot to create Muslim infertility (33). Others say that the West is supplying Yemen with inadequate vaccinations (34). People in DFA regions, on the other hand, do not see COVID-19 as a danger (32).

### Policy issues

Yemen and Somalia do not contain any policy issues regarding COVID-19 vaccination, therefore policy is not a parameter of VH in these countries. In Yemen, the cost of vaccinations as well as doubts regarding quality and safety were primary reasons for VH, as stated by Bitar et al. (11) and Bin Ghouth et al. (13). In Somalia, the major cause of VH was fear of side effects, followed by concerned regarding inefficacy, as mentioned by Ahmed et al. (10). However, issues regarding allocation of vaccinations in Palestine may play a major role in VH (35), due to COVID-19 vaccines shortage (36). As stated by Kateeb et al. (23), one reason for VH in Palestine was availability of the vaccines. In Afghanistan, the new government has banned COVID-19 vaccinations in some regions, therefore possible repercussions of being inoculated could be a major reason for VH (37). In Syria, vaccine discrimination between civilians in government areas could be a source of VH, as many people with influence may receive unfair advantages (38). Mohamad et al. (17) mentions a cause of VH to be lack of availability amongst L-LMICs.

## Discussion

This present narrative review provides the most up-to-date information on vaccination reluctance in conflict settings. This is especially crucial in conflict zones where misinformation flourishes and people are unaware of the potentially disastrous implications of it. Furthermore, decreasing immunity as well as mutations in viral strains of COVID-19 like i.e., Omicron and delta variants further adds to the dilemma and spread of the infection.

The findings of this research revealed a broad range of willingness to attain the COVID-19 vaccine in different conflict zones. Vaccine reluctance is at or above 30% in nearly all conflict zones. According to studies conducted in Yemen and Syria, the populace is more than 60% hesitant to vaccinate owing to conflict and instability (refer to Table 1). There has only been one study conducted in Somalia which found that 23.2% of the population is reluctant to receive vaccinations, which could easily be overcome by addressing the factors associated with vaccine hesitancy, such as fear of side effects and misinformation about the vaccine being fabricated (10). Afghanistan is in a deepening humanitarian crisis, already facing enormous issues such as food insecurity, intercommunal conflict flare-ups, floods, and a weakened health system, with the COVID 19 pandemic adding to the load. Nemat et al. study revealed that more than one-third (37%) of individuals were unwilling to receive the COVID-19 vaccination. Those unwilling were influencing others with unauthentic opinions about the COVID-19 vaccine, leading to an increase in vaccination refusal. This, in turn, made achieving herd immunity to combat COVID-19 infection in Afghanistan challenging (19). Results from a survey conducted in Palestine between December 2020 and January 2021 revealed a 37.8 per cent inclination to be vaccinated among the participants (22).

The World Health Organization (WHO) estimates that the COVID-19 pandemic killed over 15 million people worldwide, a tragic number that could have been avoided by making vaccinations available to all and investing in health care systems. Armed conflict has a significant impact on healthcare systems, causing infrastructure to be destroyed or overlooked and impeding supply chain operations (39). Østby et al. found that conflict exposure, especially its severity during the first year of life, had a statistically significant and unfavorable influence on vaccination rates (40, 41). According to earlier research, vaccination supplies for 2022 have improved significantly over the last few months, with just 14% of people in low-income nations having received at least one dose as of March 2022 (41). It is now obvious that COVID-19 immunization rates in low-income countries have been extremely lacking. The figures are especially low in nations suffering from violent conflict and humanitarian crises. Only 7.5 percent of the 283 million people in the world's ten “most unstable” states are completely vaccinated against COVID-19. Violence hinders efforts to give vaccinations in several of these countries (41).

There are several causes for vaccine reluctance in conflict settings. Most typically, it was due to a lack of awareness about the vaccine, fear of catching the virus after vaccination, and suffering adverse effects, as well as trust in one's own immunity. Despite, Yemen's condition is extremely precarious, as it is already undergoing the world's biggest humanitarian disaster, and the COVID-19 epidemic has just aggravated the situation for the state (42).

Improving immunization rates in conflict zones is critical. Many unstable states lack the funding, qualified staff, and healthcare systems needed to efficiently deliver vaccination, such as ultra-cold freezers and electricity to keep the doses viable. These challenges are exacerbated in countries like Syria, Gaza, and Yemen, where conflict has devastated hospitals, highways, and other critical infrastructure. People in conflict zones or other regions where state services are restricted may lose track of immunization records that they have already obtained. The governments should increase their mass vaccination promotion on both mainstream and social media. Health professionals should make efforts to minimize misconceptions and rumors. Effective campaigns should be launched to inform refugees and migrants about when and where to receive the vaccination, as well as its advantages and safety. Understanding the hurdles *via* research performed in conflict zones, and developing targeted, evidence-based methods and communication plans will be the most effective strategy in eradicating vaccine hesitancy. Capacity-building and training to develop and extend the capability of health systems, as well as health literacy awareness programs for refugees and migrants is crucial (43). Overall, the utmost priority must be given in addressing the lack of awareness most conflict zones face regarding the COVID-19 vaccine. This can be conducted by providing vaccine related information to the masses through campaigns, social media and even door to door service. It can be further enforced on certain groups of individuals such as students and workers of any career that COVID-19 vaccination has become a necessity when entering school or work premises. To gain the trust of the hesitant majorities, vaccinated candidates must be encouraged to assure people in their surroundings that the vaccine is safe and important. The root causes of VH also lie specifically for each country. While complete changes may require time, it can be ensured that COVID-19 vaccine's hesitancy can be reduced through genuine efforts by individualistic and authoritarian approaches.

In addition, female healthcare workers must stop being harassed, and access to health services must be assured. To ensure the security of Afghan civilians, a national strategy should be developed to control the spread of the pandemic (44). Furthermore, governments like those in Gaza cannot afford the costs of public awareness campaigns due to existing economic instability. To address this scenario, these governments should establish organizations that have their work done through social media surveys and randomized tests, allowing us to understand people's attitudes about COVID-19 and vaccination intentions (45).

### Study limitation

Limitations include potential sample bias in numerous studies that formed the foundation of the current narrative review. Second, the diverse time points at which these surveys were done can confound the interpretation of their results, particularly concerning the timing of COVID-19 vaccine deployment in each country. Furthermore, results might be affected due to various criteria used to define COVID-19 vaccine acceptability. Moreover, the drivers of vaccination hesitancy highlighted in this review were those discovered in most research that primarily used online questionnaires. While this was unavoidable given the COVID-19 pandemic's lockdowns and travel bans, demographic groups with restricted internet access, such as elderly persons, may be overlooked. Lastly, despite a comprehensive search strategy and screening were done meticulously to the best of our knowledge. Conference proceedings as well as articles not in English were excluded. Hence, our narrative review does not guarantee the inclusion of all the relevant studies for the purpose reported.

## Conclusion

This narrative review shows at least three-tenths or more of vaccine hesitancy is observed among conflict settings. Conceivably, vaccine hesitancy was mostly due to a lack of appropriate knowledge about the vaccine, lack of available resources, and believing misleading rumors about the vaccine. Thus, considering the massive amount of reluctance among people residing in conflict zones, the need to take effective measures to combat vaccine hesitancy is undoubtedly apparent. This can be accomplished by carrying out mass vaccinations by the government and proper health education regarding vaccines, thereby attenuating rumors that exacerbate the fear of adverse effects. Therefore, the approach described in this article to combat vaccine hesitancy can be implied to increase vaccination rates and significantly alleviate R_0_ for COVID-19 cases across the globe, subsequently curbing the pandemic in a pragmatic way.

## Author contributions

Conceptualization and project administration and supervision: AS and ME. Methodology: AS. Visualization: AS and AA. Writing—original draft: AS, P, AA, SA, and KQ. Writing—review and editing: AS, ZR, RF, and ME. All authors contributed to the article and approved the submitted version.

## Conflict of interest

The authors declare that the research was conducted in the absence of any commercial or financial relationships that could be construed as a potential conflict of interest.

## Publisher's note

All claims expressed in this article are solely those of the authors and do not necessarily represent those of their affiliated organizations, or those of the publisher, the editors and the reviewers. Any product that may be evaluated in this article, or claim that may be made by its manufacturer, is not guaranteed or endorsed by the publisher.
